# Physically demanding occupations among females and sex-related differences to develop osteoarthritis of the hip: a systematic review and meta-analysis

**DOI:** 10.1186/s12995-024-00415-8

**Published:** 2024-05-06

**Authors:** Susanne Unverzagt, Annekatrin Bergmann, Kathleen Denny, Thomas Frese, Selamawit Hirpa, Johannes Weyer

**Affiliations:** 1 Institute of General Practice and Family Medicine, Martin Luther University Halle-Wittenberg, Faculty of Medicine, Halle (Saale), Germany; 2 Department of Occupational Medicine, Martin Luther University Halle-Wittenberg, Faculty of Medicine, Halle (Saale), Germany

**Keywords:** Occupational, Hip, Osteoarthritis, Workplaces, Musculoskeletal, Gender medicine

## Abstract

**Background:**

Hip osteoarthritis (HOA) is a leading cause of disability increasing with age and is more prevalent in women and in various physically demanding occupations. This systematic review identifies and summarises occupational exposures for women in physically demanding occupations and discusses sex differences and consequences.

**Methods:**

In this systematic review, we searched various electronic databases for reports published between date of database inception and October 2022. We included cohort studies and case-control studies that assessed the association between exposure to physically demanding occupations and the development of HOA. We then assessed the methodological quality of selected studies, extracted relative effects, compared the risk for women and men and meta-analytically reviewed the effects of physically demanding occupations. All steps were based on a study protocol published in PROSPERO (CRD42015016894).

**Results:**

We included six cohort studies and two case-control studies in this systematic review. These studies showed a considerably increased risk of developing HOA in both sexes. Women working in traditionally female-dominated occupations such as cleaning, sales, catering, childcare and hairdressing that are physically demanding, have a higher risk of developing HOA than men in similarly physically demanding occupations. Conversely, in traditionally male-dominated occupations with a high heterogeneity of work activities, such as agriculture, crafts, construction, as well as in low-skilled occupations, the risk was higher for men. One exception are health occupations, which are grouped together with a wide range of other technical occupations, making it difficult to draw conclusions.

**Conclusions:**

Existing studies indicate an association between various occupations with a high physical workload and an increased risk of developing HOA. Occupational prevention and individual health promotion strategies should focus on reducing the effects of heavy physical workloads at work. The aforementioned as well as early detection should be specifically offered to women in female-dominated occupations and to people working in elementary occupations.

**Supplementary Information:**

The online version contains supplementary material available at 10.1186/s12995-024-00415-8.

## Background

Osteoarthritis (OA), a degenerative joint disease, is one of the leading causes of disability worldwide [1, 2]. The disease is defined by pathological changes in the hip. This includes the articular cartilage, subchondral bone, ligaments, capsule, synovium and periarticular muscles [3]. The pain and progressive loss of mobility in the affected joint undermine the quality of life and productivity of patients. Apart from individual well-being, OA has a significant economic impact due to reduced work productivity, absenteeism resulting from sickness and disability [4–6], and treatment costs [7].

OA is caused by the degeneration of the articular cartilage and changes in the subchondral bone structure that cause pain and limit joint mobility [8, 9]. According to clinical guidelines, the diagnosis of hip osteoarthritis (HOA), also known as coxarthrosis, is based on the triad of joint pain, limited mobility, and radiographic findings [10]. Radiographic findings include narrowing of the joint space, osteophyte formation, and subchondral sclerosis [9]. The most commonly affected peripheral joints are the hips, knees, and hands [9].

The global prevalence of HOA is estimated to be 8.6 % (95 %-confidence interval [CI] 4.8 to 13.2), with Europe having the highest regional estimate at 12.6 % (95 %-CI 7.2 to 19.2) [11]. The past decades saw a global increase in the age standardised incidence rate (ASIR) of HOA from 17.0 (95 %-CI 12.7 to 22.0) in 1990 to 18.7 per 100.000 people (95 %-CI 14.0 to 24.2) in 2019. In the same period, in Western Europe, the ASIR increased from 33.4 (95 %-CI 24.9 to 43.0) to 38.4 per 100.000 people (95 %-CI 28.4 to 49.7) [12]. Risk factors for HOA can be classified into three categories: biological, lifestyle-related, and occupational. The most common determinants are female sex [13], old age [8], high body mass index (BMI) [14], metabolic disorders [8], and genetics [9]. The prevalence of HOA generally increases with age, and most cases are diagnosed in individuals over 60 years old [5].

Various studies show a positive relationship between prolonged lifting and carrying of heavy loads or physically demanding work in general, and the risk of developing HOA [15–17]. In a previous systematic review, we found that there is a strong association between occupational exposure to other physically demanding ergonomic risk factors, such as force exertion, tiring posture, repetitiveness, lifting, kneeling and/or squatting, and climbing, and HOA among men [18]. Although there are known differences between men and women in the prevalence of musculoskeletal disorders and pain [19], resulting healthcare use, and work disability [5, 20], there is limited and inconsistent evidence linking occupational exposures to HOA [13, 21, 22]. Gignac et al. [23] investigated the evidence for an increased risk of (HOA) associated with various occupational activities for both women and men.

The aim of this systematic review is to determine the association between work-related exposures in occupational sectors with high physical workloads and exposure to ergonomic risk factors for females and the risk to develop HOA. We wanted to compare the risk of females and males and discuss gender-specific differences and consequences. Gender was categorized as female or male and refers to the socially constructed roles and behaviours [24].

## Methods

This systematic review was registered in PROSPERO (CRD42015016894) prior to its commencement. It adheres to the PRISMA guidelines [25] and provides additional evidence on the association between physically demanding occupations and the development of HOA, building on our recent publications [15, 16, 18].

### Systematic search

This systematic review is based on the strategy used in our recently published review [18]. We updated our systematic search strategy by adding specifically female-dominated occupations. We searched Medline (Ovid), Embase (Ovid), the Cochrane Library, CINAHL and the Health and Safety Executive (HSE)-Line for studies published between database inception and October 2022. Our search strategy was based on a combination of controlled vocabulary and key words describing the association between occupational exposure and the development of HOA. All references were imported into a bibliographic reference management programme (Endnote).

### Eligibility criteria

The focus of this review is to determine the association between occupational exposures in females and the development of HOA. We used the following criteria to define population, exposure and outcomes for question:

Population: Adult persons (≥ 18 years at diagnosis).

### Exposure

Former or current employment in physically demanding occupations that frequently involve lifting, exerting force, postures, repetitive tasks, kneeling or squatting, and climbing activities of varying levels of intensity and duration where results on the association between occupational physical demands’ and development of HOA were reported in at least two studies.

### Outcomes

To be included studies had to report a diagnosis of HOA due to occupational physical demands’based on

- classification of HOA through clinical and radiological criteria (American College of Rheumatology criteria [10] or according to Kellgren and Lawrence [26] through radiological scoring systems,
- total hip replacement subscales measuring hip pain, stiffness or reduced physical function,
- hip pain or
- Reported cases of disability, pension or sick leave due to a diagnosis of HOA in registry data.

We excluded studies with HOA due to non-occupational physical demands (e.g. due to sport or hip deformities such as hip dysplasia).

Study design: We included full-text publications in German or English language from 1990 until October 2022 of cohort studies, case-control studies or analysis of data from registry data and cross-sectional studies with relevant data on exposure to occupations for at least 10 years to assure a causality.

### Study selection

Two independent reviewers checked the titles and abstracts of all references identified in our systematic search from different sources. They then read the full text of potentially eligible studies, extracted data and assessed the quality of the included studies. In case of disagreement, a third reviewer was consulted to reach a consensus.

### Data extraction

We extracted information to characterise the reviewed studies (design, country and time of recruitment), study population (inclusion criteria, number of participants, age and gender), exposure and reference groups (including levels of exposure with their duration and intensity) and outcome (with precise diagnostic criteria). We extracted all occupations for which results were reported for women and selected those occupations for which effect measures for the investigated association were reported for women in more than two studies. For these occupations, we extracted the percentage of women to distinguish between female and male-dominated occupations, the number of participants and events per group and the adjusted effect sizes with their 95 % CI for both women and men.

### Quality assessment

The criteria used in our research question were based on Bergmann et al. 2017 [15], who applied the Newcastle-Ottawa Quality Assessment Scale [27] and the Cochrane Handbook [28]. The assessment criteria were developed separately for case-control and cohort studies and resulted in a summarized quality score. For cohort studies, we assessed the representative selection of exposed and non-exposed participants, the validity and accuracy of exposure and outcome ascertainment (diagnosis of HOA), and the methods used to ensure comparability between the exposed and non-exposed groups. For case-control studies, we assessed the representativeness of the selection of cases and controls, validity and accuracy of exposure assessment, as well as the methods used to ensure comparability between cases and controls. This assessment yielded a maximum of 19 points for cohort studies and up to 15 points for case-control studies.

### Data synthesis

The association of occupational risks and development of HOA in occupations with heavy physical strain was compared to less-exposed reference groups using various effect measures. All effect measures (odds ratios, relative risks, hazard ratios, standardised hospitalisation ratios) were interpreted as relative risks (RR) due to the low prevalence of HOA. If more than one effect estimator was reported in a study, we pooled comparable results from studies that corresponded best with our research question. We selected the estimator based on diagnostic criteria with the best validity according to Bergmann et al. 2017 [15] and the highest or longest exposure with a sufficient sample size. We used the reported adjusted results with their corresponding 95 %-CI.

The effect estimators of different studies and their 95 %-CI were synthesized with the random effects model using RevMan 5.3 [29]. We chose this model due to differences in measurement of exposure, outcome, study design and effect measures. Reported RRs greater than 1 describe a higher risk in occupations with heavy physical strain compared to the reference group. We judged the consistency of results of different studies based on the I^2^ value and interpreted heterogeneity as small (I^2 ^< 30 %), moderate (30 to 60 %) or substantial (I^2^> 60 %). We did not discuss the pooled results in cases of substantial heterogeneity between study results, different conclusions of the studies or clinical heterogeneity in severity of physical demands. To investigate clinical heterogeneity between treatment effects of individual studies, we calculated subgroup analyses for differences in exposure, criteria to diagnose HOA and study design. We quantified the influence of sex with ratios of RRs comparing the RR of females and males. Ratios over 1 describe a higher risk of females.

## Results

**Fig. 1:**
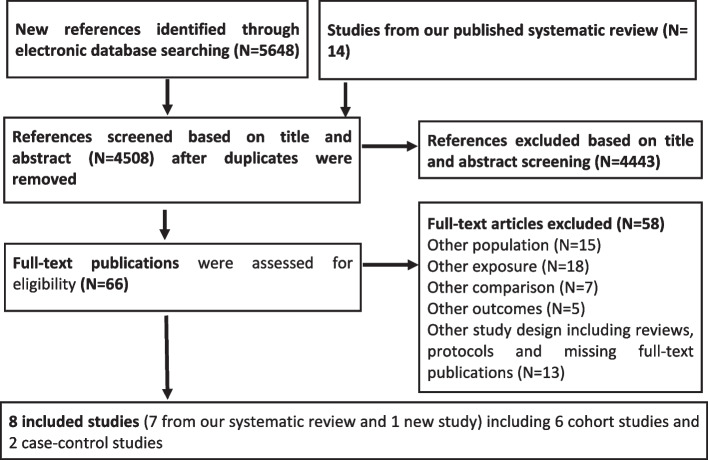
PRISMA flow chart to describe identification and selection of included studies

Using our adapted search strategy, we identified a total of 5648 new references and included 14 studies from our recently published systematic review [18]. We screened 66 reports for relevance and excluded 58 studies as shown in Fig. 1. The excluded studies primarily investigated men (*N*=15), did not report sex-specific effect estimates on occupational exposure effects (*N*=18), did not compare exposed and unexposed groups (*N*=7), or did not report outcomes (*N*=5). We also excluded studies with other study designs including systematic reviews, protocols or cross-sectional studies with short follow-up periods (*N*=11). Additionally, we excluded studies that did not have a final full-text publication (*N*=2).

Ultimately, we included eight eligible studies including six cohort studies [13, 22, 30–33] (Table 1) and two case-control studies (Table 2) [34, 35]. Table 1 and table 2 summarize the characteristics of these cohort and case-control studies. No cross-sectional studies with at least 10 years of occupational exposure to ascertain the association between occupational exposure and the development of HOA were identified.

**Table 1:** Characteristics of eligible cohort studies on the association between physically demanding occupations and the development of osteoarthritis

***Study*** *Country*	***Population***	***Exposition (occupational groups)*** ***vs. Reference***	***Outcome (HOA)***	***Quality score*** *(S/E/C/D)*
***Åkesson 1999*** *Sweden* [30]	*Female dentists working in the public dental care service* *N=120* *Age: median dentists: 40 (range 28-57) years; dental assistants: 37.3 (range 25-37) years, median dental hygienists: 41.5 (range 28-52) years* *Female: 100 %*	*Dentists/ dental hygienists (from the same country, with a similar organization and working environment, matching age)/ dental assistance (same population, matching to dentists (sex and age ± 5 years)) vs.* *female medical nurses (aged 41.7 (26- 60) years) chosen of varied and physically work load*	*Musculoskeletal symptoms, pain rating via Nordic questionnaire* *Clinical diagnostic examination using a standardized protocol for diagnoses in the hip region* *Follow-up: 5 years*	***8**** (2/1/2/3)*
***Andersen 2012*** *Denmark* [31]	*Population-based register data of 5 occupational groups, ≥ 16 years (1981-2006)* *N=2 117 298* *Age (median, IQR); % Women:* *Farmer: 44 (32-59) years; 55 132 (25.4 %)* *Construction worker: 38 (27-52) years; 38 485 (7.9 %)*^*a*^ *Healthcare assistants: 39 (28-51) years; 411 580 (82.9 %)*^*a*^ *Office workers: 36 (25-51) years; 595 685 (65.3 %)*^*a*^	*Register data of the income register in the basis on work skills/position and education (1981-2006)* *Floor layers/bricklayers/pavers, construction workers, farmers, healthcare assistants) vs.* *office workers*	*Surgically treated HOA (ICD, 1996 to 2006)* *Follow-up: 8.1-9.2 years*	***15**** (4/3/3/5)*
***Hubertsson 2017*** *Sweden* [13]	*Population-based register data of included occupational groups, 40-70 years (2008-2012)* *N=165 179* *Age (mean): 55 years* *% Women: 96 272 (58 %)*^*a*^ *Health care: 45 211 (90 %)* *Child care: 16 308 (93 %)* *Cleaning: 8043 (83 %)*	*Register data with classifications of current jobs (12/2012)* *7 occupational groups at a higher risk of HOA including more female-dominated occupations vs.* *business and administration (e.g. business and finance professionals, mathematicians, statisticians, computing professionals, secretaries and numerical clerks)*	*Sick leave and disability pension due to HOA (ICD, 2007-2012)* *Follow-up: ≤ 6 years*	***12**** (4/3/2/3)*
***Johansson 2018*** *Sweden* [32]	*Patient register data (1987), ≥ 35 years; Follow up until 2002* *N= 3 560 496 total* *N=97 136 farmer* *% women: 24 284*^*a*^* (25 %)* *Age 58.3 ± 12.2 years*	*Population and Housing Census 1975 an 1985 provided self-reported profession based on Nordic class profession (NYK)-farmers, foresters, market gardeners (ISCO Code 401)* *vs. all other occupations*	*HOA* *Follow up until 2002 (15 years), death or emigration*	***11(2/2/4/3)***
***Solovieva 2018*** *Finland* [22]	*Population-based register data from a random sample of persons with gainful jobs, 30-60 years (01/2005)* *N= 1 135 654* *Age (mean): 43.8-47.5 years in occupational groups* *% Women: 561 037 (49.4 %)*^*a*^ *Professionals: 57 226*^*a*^* (42.6 %)* *Agricultural and fishery workers: 17 953*^*a*^* (34.6 %)* *Construction worker, electricians and plumbers: 2244*^*a*^* (4.2 %)* *Environmental officers and nurses: 42 639 (88.6 %)* *Shop worker: 28 052*^*a*^* (67.6 %)* *Customer service clerks: 17 953*^*a*^* (91.8 %)* *Building caretakers, cleaners, assistant nurses, and kitchen workers: 43 761*^*a*^* (72.8 %)* *Craft worker: 4.488*^*a*^* (36 9%)* *Teaching professionals: 48 249*^*a*^* (66.9%)* *Unskilled transport, construction, and manufacturing workers: 6732*^*a*^* (25.8%)*	*Register data of longitudinal employer-employee data with classifications (12/2004)* *18 occupational groups vs.* *professionals (e.g. physical, mathematical, and engineering science professionals,* *life science and health professionals, as well as others )*	*Full-time disability retirement due to HOA (ICD, 2005-2013)* *Follow-up: 9 years*	***14**** (4/2/4/4)*
***Vingard 1991*** *Sweden* [33]	*Register sampling of persons, born 1905-1945 with the same occupation in 1960 and 1970 (1980)* *N: 250 217* *Age (range): 35-75 years* *% Women: 42 579 (17.0 %)*^*a*^ *Low exposure blue-collar workers: 24.145 (21.0 %)*^*a*^ *Farmer: 1739 (4.6 %)*^*a*^ *Waitresses and hairdressers: 7243 (99.8 %)*^*a*^ *Cleaner: 7625 (100 %)*^*a*^	*Blue-collar occupations with high (more than average) (14 for males, 5 for females) vs.* *low (less than average) exposure to dynamic or static forces acting on lower extremity*	*Hospital care for HOA (ICD, 1981-83)* *Follow-up ≥ 15 years*	*13 (4,2,2,5)*

^a^calculated by the authors

*C* comparability, *D* diagnosis, *E* exposure, *HOA* Hip osteoarthritis, *ICD* International classification of diseases (version 8-10), *n.d* no data, *S* selection, *SES* socio-economic status, *THR* total hip replacement

**Table 2:** Characteristics of eligible case-control studies on the association between physically demanding occupations and the development of osteoarthritis

**Study** Country	**Population**	**Description Cases vs. Controls**	**Exposition (occupational group) vs. reference**	**Quality score** Total (S/A/E)
**Elsner 1995** Germany [34]	Patients of an orthopaedic practice *N*=418 (220/198) Response cases: 60 %, control: n.d. Age (range) of cases: 30 % - > 60 years with younger controls Women (%): 189 (45 %)^a^ Nurses and kindergarten teacher: 18 (100 %)^a^ Health care: 10 (100 %)^a^ Retail worker: 27 (71 %)^a^ Workers in hotels, gastronomy and households: 22 (61.1 %)^a^ Occupations in textile industry: 13 (100 %)^a^ Cleaner: 8 (61.5 %)^a^ Hairdresser: 6 (100 %)^a^	**cases:** patients with hip symptoms and radiographic signs of HOA vs. **controls:** persons without hip symptoms from a general practice and an ophthalmologist, church community 1989-1993	**Questionnaire** on occupational history, occupational groups named by ≥ 5 persons vs. all other men or women	**5** (1/2/2)
**Franklin 2010** Iceland [35]	All patients and their first-degree relatives ≥ 60 years Response cases: 33 % *N*= 2490 (1408/1082) Age: cases: 71-75/ controls: 71 years Women (%): 1424 (832/592) (57.2 %) Managers and professionals: 115 (54.5 %) Farmer: 242^a^ (46.7 %)^a^ Technicians and associate professionals: 196^a^ (63.8 %)^a^ Service and shop worker: 246^a^ (78.8 %)^a^ Craft worker: 196^a^ (45.7 %)^a^ Operator and unskilled labour: 162 (53 %)	**cases:** all patients with TKR or THR due to OA, at surgery (1967-1998) vs. **controls:** first-degree relatives 1998	**Questionnaire** on occupational history, longest held occupations coded into 8 groups, 6 groups vs. manager and professionals (e.g. teachers, doctors, nurses)	**10** (3/4/3)

^a^calculated by the authors

*A* adjustment, *E* exposition, *HOA* Hip osteoarthritis, *n.d* no data, *S* selection, *THR* total hip replacement

### Participants

The eight included studies were conducted in the Scandinavian countries [13, 22, 30–33], Germany [34], or Iceland [35]. In the six cohort studies the duration of follow-up varied from 5 to more than 15 years. Most participants were people of working age (20 to 70 years), with a mean age of 37 to 58 years at the time of the examination. The gender distribution varied among occupational groups. On the one hand more women working in health care, cleaning, sales, catering, childcare, hairdressing and clerical work, and on the other hand more men working in agriculture, craft trades, construction, elementary occupations or as managers and in executive positions (see Table 1 and Table 2).

### Exposure

Information on occupation was mainly based on registry data [13, 22, 31–33] in the Scandinavian countries, and on occupational titles [30] or occupational history questionnaires [34, 35] in Germany and Iceland. Some studies grouped different occupational categories, others were more precise and distinguished between levels or durations of exposure. We identified six female-dominated occupations and three male-dominated occupations that reported results for women. The following female-dominated sectors, known to be physically demanding, were considered and compared with less-demanding occupations:

- Health care (6 studies): Four of these studies pooled similarly demanding occupations in the health sector [13, 22, 30, 31, 34], others pooled nurses and nursery school teachers [34], technicians and associate professionals including office clerks and nurses [35], or environmental officers and nurses [22]. One study compared the risk between dental workers and medical nurses [30] (Supplementary Table S 1).
- Cleaning (4 studies): Studies grouped cleaning occupations [13, 33, 34] or pooled the risk of building caretakers, nursing assistants and kitchen workers [22] (Supplementary Table S 2).
- Sales (3 studies): Studies reported the risk of working in service, or as a retail- or shop worker [22, 34, 35] (Supplementary Table S 3).
- Gastronomy (3 studies): All of these studies combined different occupations with comparable exposures, such as employees in restaurants and hotels [34], kitchen workers, janitors, cleaners and nursing assistants [22] or waiters and hairdressers [33] (Supplementary Table S 4).
- Child care (3 studies): These studies covered all facets of childcare including nursery school teachers [34], childcare in general [13] and teachers [22] (Supplementary Table S 5).
- Hairdressing (2 studies): Both studies included hairdressers [34] or pooled hairdresser and waiting staff [33] (Supplementary Table S 6).

The following occupational male-dominated physically demanding occupations were considered:

- Agriculture, fishery or forestry (5 Studies): Most studies focused on farmers [31–33, 35], one study summarised the exposure of farmers and forest workers [34] and one study examined the exposure of agricultural and fishery workers [22] (Supplementary Table S 7).
- Craft work (3 studies): One study included craft workers without further information [22], another study included occupations in the textile industry [34] and the last study included craft workers and related trades [35], with the most common occupations being fish processing for women and carpentry or construction for men (Supplementary Table S 8).
- Construction (2 studies): Both studies included female construction workers [22, 31]. One study also included electricians and plumbers in the exposure group [22] (Supplementary Table S 9).
- Unskilled or elementary jobs (2 studies): Both studies examined the risk of HOA in women in these jobs such as unskilled transport, construction and factory work [22] (Supplementary Table S 10).

The studies compared the risk of developing HOA in physically demanding occupations with the risk in less or less physically demanding occupations including office workers, managers and professionals (see Table 1 and Table 2).

### Diagnosis

Follow-up periods between exposure and diagnosis were mainly available from cohort studies and ranged from 5 years [30] to over 15 years [33]. Various diagnostic criteria were used in the studies, including disability pension due to HOA [13, 22], implantation of a total hip replacement (THR) implantation [35], surgical treatment of HOA [31], hospitalisation due to HOA [33], clinical or radiological diagnostic criteria [31, 33, 34], sick leave due to HOA [13], or musculoskeletal symptoms in the hip [30]. We assumed with the diagnosis for disability pension due to HOA, THR and radiological imaging had a high level of validity when compared to a clinical diagnosis and hip pain (see Table 1 and Table 2).

## Quality assessment

### Cohort studies

The six cohort studies scored between 8 and 15 points out of a maximum of 19 achievable quality points. Four studies met almost all quality criteria for selection of participants [13, 22, 31, 33] and one study was downgraded as it recruited participants from solely selected practices and due to low diagnostic validity [30]. None of the studies could provide accurate and reliable quantitative data on the frequency and duration of occupational exposure. All studies, except one [30], adjusted results for age; two of them used other important confounders (body mass index (BMI) or education and factors of physical workload [13, 22] to ensure comparability. Only two trials scored almost full points for the diagnosis of the outcome, taking validity and follow up into consideration [31, 33].

### Case-control studies

The two case-control studies received 10 [35] and 5 [34] quality points on a scale of 0 to 15. Both were downgraded because of deficiencies in the selection (selection of controls, low response rate and validity of data collection), and accuracy of exposure measurement. One study was also downgraded because some comparative hypotheses mentioned in the methods section were not reported with quantifiable data [34]. One study received full quality points for comparability and adjustment, adjusting for age and body weight, and also full points for an adequate case definition by including people with a THR [35].

## Study results

### Female-dominated occupations

#### Health care

**Fig. 2:**
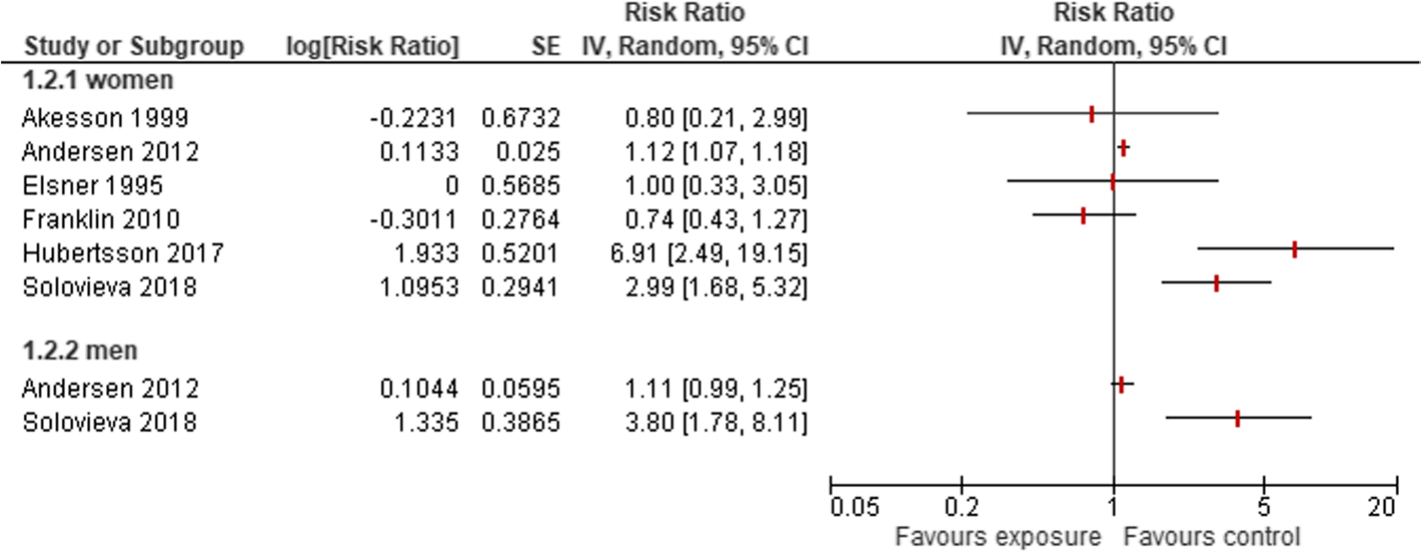
Risk of developing HOA owing to occupations in health care

Six studies [13, 22, 30, 31, 33, 34] included approximately 500,000 women employed in, with 2935 of them being diagnosed with HOA (Supplementary Table S 1). Three studies stated an elevated risk of diagnosis of HOA for women working as health care-assistants, nurses, dental personal or midwives. Results varied from a low risk (Franklin 2010, Ǻkesson 1999) to a 6.9-fold risk of women in healthcare receiving a diagnosis of HOA (Hubertsson 2017), resulting in a substantial heterogeneity of results (Fig. 2). Especially the exposure of the higher and lower physically demanding groups are comparable in the studies of Ǻkesson 1999 and Franklin 2010. However, Andersen 2012 and Solovieva 2018 reported an increased risks for both men and women. Andersen et al. (2012) reported a comparable increase for women and men working as health-care assistants (RR 0.80; 95 %-CI 0.21 to 2.99 vs. 1.11; 95 %-CI 0.99 to 1.25), whereas Solovieva 2018 reported a 0.79 lower risk for women compared to men working as nurses and environmental officers compared to professionals (RR 2.99; 95 %-CI 1.68 to 5.32 vs. 3.80; 95 %-CI 1.78 to 8.11) Fig. 2).

#### Cleaner

**Fig. 3:**
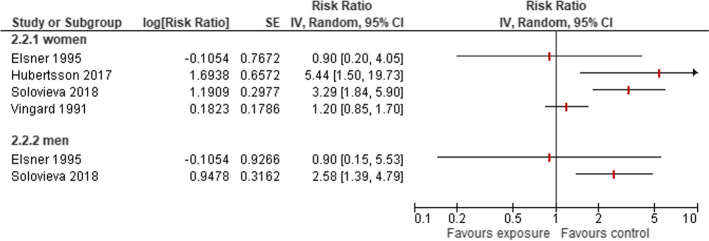
Risk of developing HOA owing to occupations in cleaning

Four studies [13, 22, 33, 34] included approximately 59,000 women working in cleaning. Of them, 248 were diagnosed with HOA (Supplementary Table S 2). The studies showed substantial heterogeneity in their results (I^2^=76 %). One study (Elsner 1995) stated no difference in the risk between cleaners and other women, whereas three studies stated an elevated risk for cleaners. This risk increase was estimated between a 1.2-fold risk for hospitalisation (Vingard 1991) and a 5.4-fold risk (RR 5.4; 95 %-CI 1.5 to 19.7) for disability pension due to HOA) in Hubertsson 2017 (Fig. 3). Two studies (Elsner 1995, Solovieva 2018) reported risks for men and women with a comparable risk in Elsner 1995 and a 1.27 higher risk increase for women (RR 3.3; 95 %-CI 1.8 to 5.9) compared to men (RR 2.6; 95 %-CI 1.4 to 4.8).

#### Sales

**Fig. 4:**
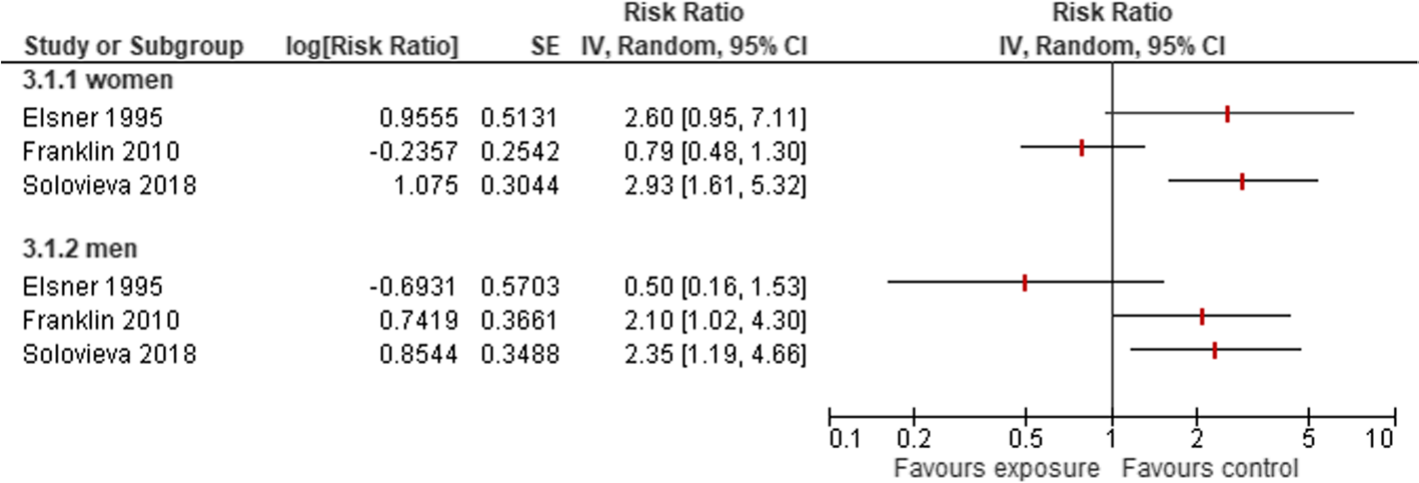
Risk of developing HOA owing to occupations in sales

Three studies [22, 34, 35] reported a diagnosis of HOA in 181 of 46,000 women working in sales (Supplementary Table S 3). Two of these studies stated an elevated risk with a nearly threefold risk (RR 2.9; 95%-CI 1.6 to 5.3) for HOA or disability pension due to HOA for shop workers (Solovieva 2018, Elsner 1995) (Fig. 4) and a 1.2 or 5.2 fold higher risk for women compared to men. The research by Franklin et al. (2010) did not state this high risk for women, even though the control group included women who were highly exposed due to their work as nurses.

#### Gastronomy

**Fig. 5:**
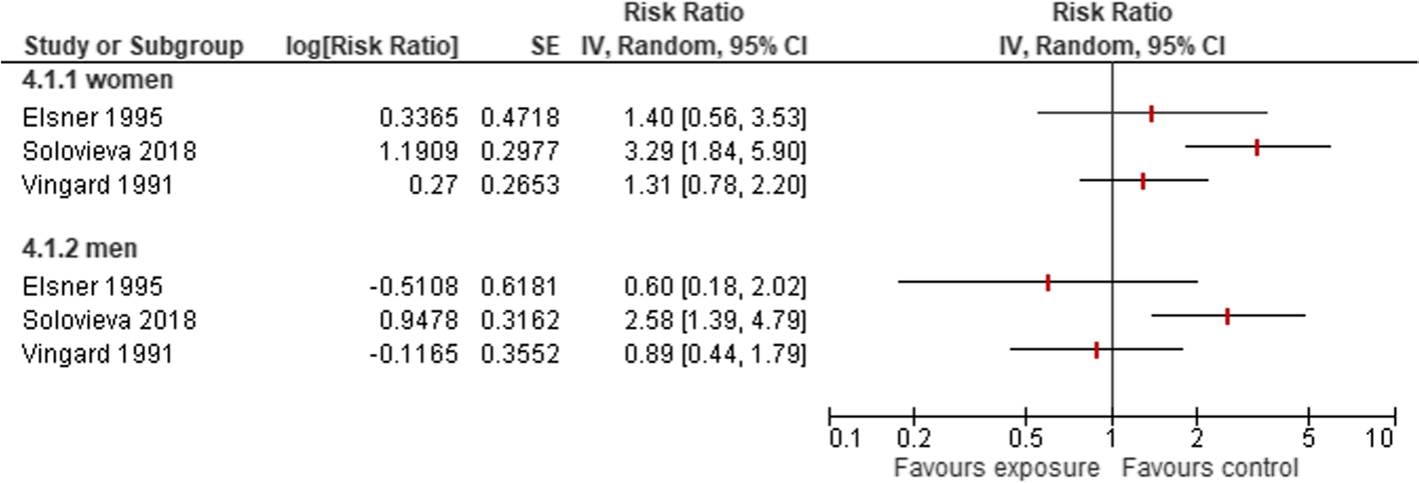
Risk of developing HOA owing to occupations in gastronomy

Three studies [22, 33, 34] included 51,026 women working in gastronomy as kitchen workers, waiters or bartenders. 226 of them were diagnosed with HOA (Supplementary Table S 4). These studies summarized very different occupational groups with resulting substantial heterogeneity of their results (I^2^= 65%). But all studies stated an elevated risk of developing HOA (Fig. 5) and a substantial higher risk for women by factors 1.3 to 2.3 compared to men in all studies.

#### Child care

Three studies [13, 22, 34] included 62892 women working in child care, as kindergarten teachers or teaching professionals. Of them, 85 were diagnosed with HOA (Supplementary Table S 5). Differences in occupational groups resulted in a moderate heterogeneity of results (I^2^= 52 %). The pooled results stated no elevated risk of developing HOA for women (Supplementary Fig. 1). One study (Solovieva 2018) reported risks for women and men with a 2.6 fold higher risk for women (RR 1.64; 95 %-CI 0.86 to 3.12) compared to men (RR 0.64; 95%-CI 0.26 to 1.58)..

#### Hairdressing

Two studies [33, 34] included 7249 women working as waitresses or hairdresser with 43 women diagnosed with HOA (Supplementary Table S 6). The results stated no elevated risk of developing HOA with low heterogeneity of results (I^2^= 0 %). One study (Vingard 1991) reported risks for women and men with a 1.3 fold higher risk for women compared to men (Supplementary Fig. 2).

### Male-dominated occupations

Three occupational groups in craft work, agriculture, fishery or forestry, and construction were dominated by men and reported risk estimates for women and men.

#### Agriculture, fishery or forestry

**Fig. 6:**
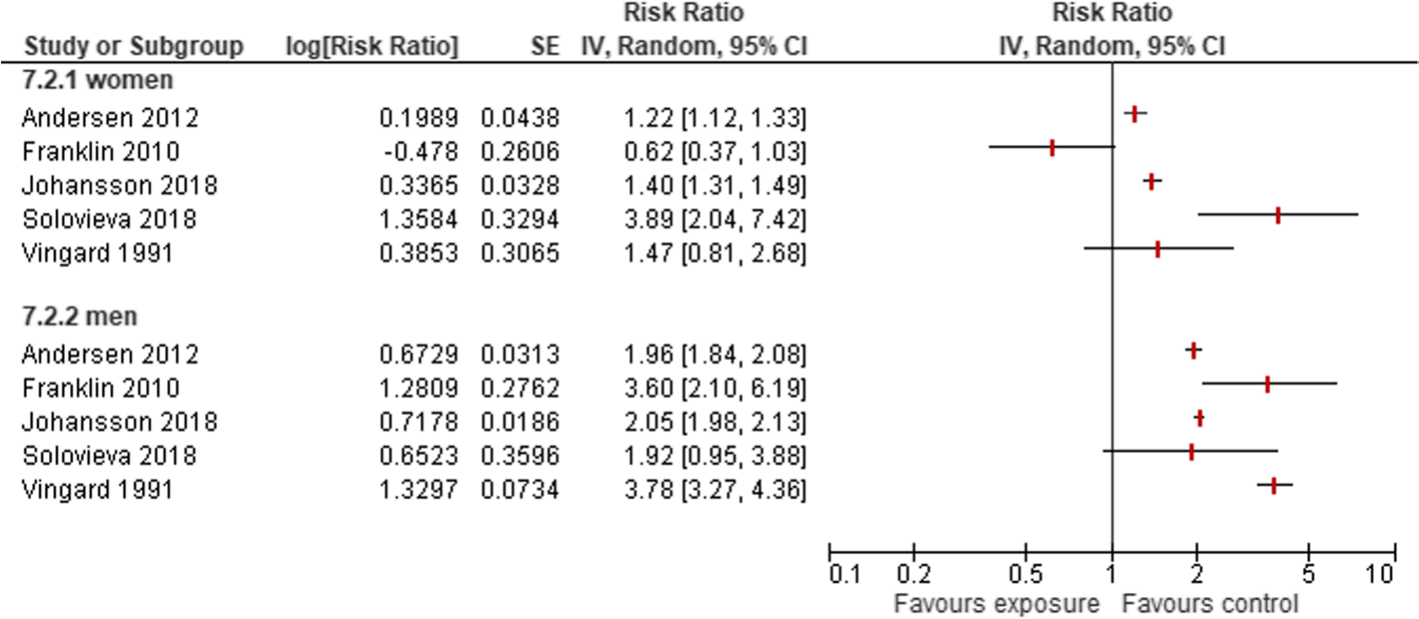
Risk of developing HOA owing to occupations in agriculture, fishery or forestry

A total of five studies [22, 31–33, 35] reported results on 99,359 women working in agriculture or fishery, and 1,926 of these women were diagnosed with HOA (Supplementary Table S 7). Aside from Franklin 2010, all studies reported an increased risk for women and men. A total of four studies reported a 1.5- to 5.8-fold higher risk to develop HOA for men (Fig. 6).

#### Craft work

Three studies [22, 34, 35] included 4697 women working as craft workers, including in the textile industry or fish processing. Of these, 93 were diagnosed with HOA (Supplementary Table S 8). The studies revealed varying risk increases for craft workers, with a nearly threefold risk observed for both women (RR 2.99; 95%-CI 1.49 to 5.99) and men (RR 2.85; 95%-CI 1.58 to 5.16) for disability pension due to HOA (Solovieva 2018) (Supplementary Fig. 3). In contrast, Franklin 2010 reported a 1.5-fold higher risk to develop HOA for men who were mostly working as carpenters or construction workers compared to women who were mainly employed in fish processing.

#### Construction

Two studies [22, 31] included 40,805 women working as construction workers, electricians and plumbers (Supplementary Table S 9). Of them, 142 were diagnosed with HOA. The studies stated very different risks with a comparable risk between men and women in Andersen 2012 and a 2.3-fold higher risk for men in Solovieva 2018 (Supplementary Fig. 4).

#### Unskilled labour

Two studies [22, 35] included 6894 women working in unskilled or basic labour (Supplementary Table S10). Of them, 75 were diagnosed with HOA. The studies stated very different risk increases with a comparable risk for both sexes in Solovieva 2018 and a 2.3-fold higher risk for men in Franklin 2010 (Supplementary Fig. 5).

## Discussion

Our results show that there are considerable occupational differences in the risk of developing HOA in both sexes. Women working in traditionally female-dominated physically demanding occupations have a considerable higher risk of developing HOA than men. These occupations include cleaning, sales, catering and hairdressing. In occupations that are traditionally male-dominated and involve physically demanding work and repetitive physical strain, men experience a higher increase in risk compared to women. These occupations include agriculture, forestry, fishing, craft trades, construction and low-skilled jobs. The exception being healthcare jobs, which studies have lumped together with other technical occupations, which are very diverse. This has made it difficult to summarise these findings. In Europe, around 76% of both men and women are exposed to at least one ergonomic risk factor [36]. The prevalence of exposure is lowest among managers and professionals (63 % and 65 % respectively), and highest among technicians, associate skilled worker professionals, clerical support workers, service and sales workers, and workers in agriculture, forestry, fishery, or craft occupations (33).According to the systematic review by Hulshof et al. [36], individuals exposed to at least one ergonomic factor for at least 2 hours per day have a more than doubled risk (OR 2.20; 95 %-CI 1.42 to 3.40) of developing hip or knee OA compared to non-exposed individuals.

The age-standardized prevalence of symptomatic HOA is higher in women than in men (0.98 % vs. 0.70 %) [37] and more women than men with a diagnosis of HOA are employed [5]. Explanations for these sex differences in prevalence as well as pain and progression include biomechanical properties, gene expression, sex hormone levels and behavior [38], as well as traditional differences in occupations and levels of physical strain in these between women and men.

### Traditional female-dominated occupations

Our findings showed that women working in cleaning, sales, catering, childcare or hairdressing are at higher risk of developing HOA than men working in the same professions. Workers in these occupations are exposed to high occupational ergonomic risks: 79% of sales and service workers are exposed to at least one of the ergonomic risk factors [39] that are associated with an increased risk of developing musculoskeletal disorders or OA of the knee or hip [36]. Physical workloads involving lifting, strenuous exertion, kneeling or squatting, demanding bent postures and repetition are typical of activities in cleaning, sales, catering, hairdressing and childcare. Repetitive activities include cashiering and loading and unloading trays. Lifting of children, cleaning products or other goods are common activities in childcare, cleaning, sales and catering. These physically demanding tasks are an integral part of daily work life and cannot be delegated. Men working in these occupations are more likely to be in managerial positions, with a much lower prevalence of physically demanding activities.

In contrast to typically male-dominated occupations, heavy lifting and carrying is not the primary exposure in female-dominated jobs. Cleaners often work in forced postures and have to use their upper body while bending forward, kneeling, or squatting. Kindergarten teachers often sit on children's chairs that are too low, and even they have to work with their upper body bent forward, kneeling, or squatting. All of these jobs involve long periods of standing and walking. For hairdressers, long standing is the most important factor. This indicates that, in addition to heavy lifting, other physically strenuous activities can also increase the risk of hip osteoarthritis.

The healthcare sector includes a variety of professions with distinct responsibilities and tasks based on their training, including medical doctors, specialized nurses, midwives, and personal care workers such as nursing assistants or nurse auxiliaries. Health-care auxiliaries in particular require a high proportion of physically demanding activities such as patient care and mobilization. When caring for patients in bed, they often work in constrained positions and are required to pull and push heavy beds. An increased risk of developing HOA was shown for these activities as a result of long-term exposure [15].

### Traditional male-dominated occupations

Occupations in agriculture, crafts and construction cover a very wide range of activities with a predominantly high exposure to ergonomic risk factors. Around 89 % of agricultural, forestry and fishery workers and 95 % of craft workers are exposed to at least one ergonomic risk factors [39]. In these occupations, men had a higher increase in risk compared to women. Traditionally, these occupations involve physically demanding activities such as lifting and carrying heavy loads, working in awkward postures and using machinery and vehicles, which cause whole-body vibrations. It is possible that only women with a hip-protective constitution choose to work and remain in these jobs, which are thought to increase risk of HOA. It is also possible that women in these occupations perform less physical demanding work.

Around 86 % of people working in elementary occupations are exposed to ergonomic risk factors [39]. This could explain the heightened risk experienced by both male and female workers in unskilled and elementary occupations, which is consistent with the findings of Solovieva et al. [22]. This study also observed a considerable decrease in exposure with higher education, which may partially explain the substantial variation between our respective studies. The level of professionalism typically increases with the duration of training. As a result, workers may have more opportunities to shape their work environment, and physically demanding tasks can be performed under more optimal conditions, such as by implementing kinesthetic concepts, that are associated with less physical strain. In the context of professionalization, the focus is often on managing issues and physically demanding activities are delegated to less qualified staff.

Other occupations that have the potential to cause damage to the hip joint include workers in mining and quarrying, firefighters and members of the armed forces. However, there is limited research on these professions, and therefore, no reliable conclusions can be drawn regarding the increased risk of HOA. This may be due to the exclusion of medical data from federal officials in regular databases. There is often a higher level of fitness and well-developed muscles due to training in military, fire and police units, which can help protect the musculoskeletal system. These professions also require good health as a prerequisite. Thus, a healthy worker effect may be observed when considering these professions.

### Limitations

The available evidence on the occupational risk of developing HOA in physically demanding occupations for women is scarce. Our systematic review is limited to studies published in English and German, and most studies were conducted in Scandinavian countries. Despite these limitations we were able to identify a number of occupations with an increased risk of developing HOA. Our study results show considerable heterogeneity among the studies that included women with variations in terms of participants, occupational groups reported as exposures, control groups, and effect estimate sizes. Some studies included participants with a mean age of less than 50 years [22, 30, 31] and had short exposure durations. This led to a low prevalence of HOA, which increases with exposure and particularly in women over 50 years of age [39, 40], possibly due to hormonal changes associated with menopause.

## Conclusion

This study shows considerable differences in the risk of developing HOA between sexes, with women in traditionally female-dominated occupations experiencing a greater increase in risk and men in traditionally male-dominated occupations experiencing a lesser increase in risk. Occupational prevention, early detection and individual health promotion strategies are needed to increase awareness and reduce the effects of high physical workloads in the workspace. These initiatives should be targeted at women in female-dominated occupations as well as women and men working in elementary occupations.

### Supplementary Information

**Supplementary Material 1.**

**Supplementary Material 2.**

## Data Availability

No datasets were generated or analysed during the current study.

## Abbreviations

ASIR: Age standardised incidence rate
BMI: Body mass index
CI: Confidence intervals
HOA: Hip osteoarthritis
OA: Osteoarthritis
RR: Relative risk
THR: Total hip replacement
