# Supplementary material for: Physically demanding occupations among females and sex-related differences to develop osteoarthritis of the hip: a systematic review and meta-analysis
Source: J Occup Med Toxicol. 2024 May 6;19:14. doi: 10.1186/s12995-024-00415-8 (PMC11071200; doi:10.1186/s12995-024-00415-8)
Supplement: Supplementary file 1 — Supplementary Material 1. [file 12995_2024_415_MOESM1_ESM.docx]

# Supplementary Figures


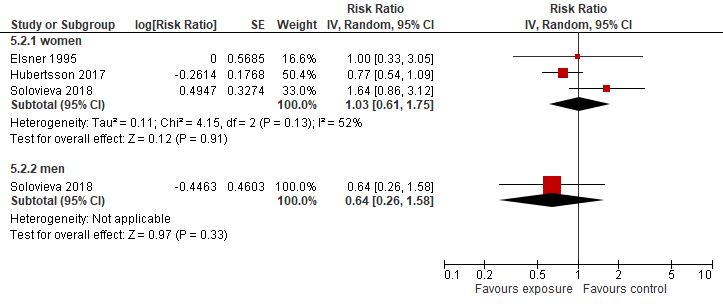


Figure 1: Risk of developing HOA owing to occupations in child care


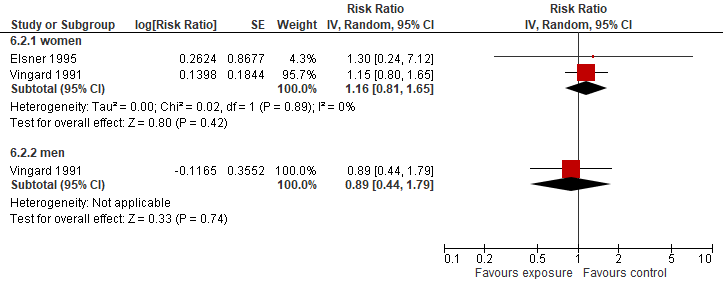


Figure 2: Risk of developing HOA owing to occupations in hairdressing


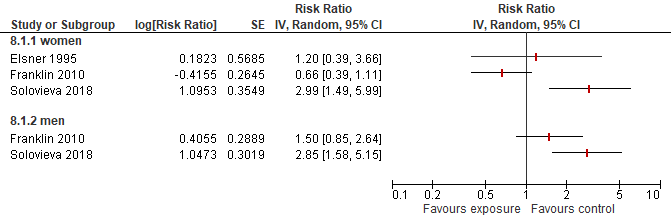


Figure 3: Risk of developing HOA owing to occupations in craft work


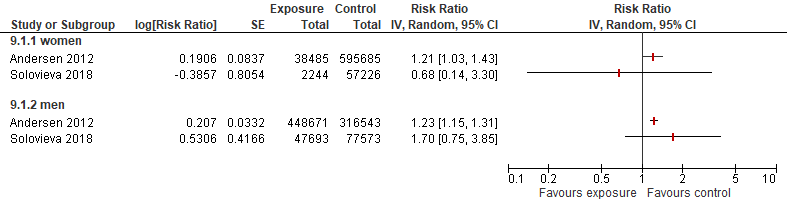


Figure 4: Risk of developing HOA owing to occupations in construction


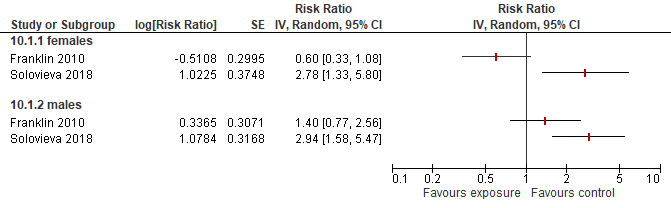


Figure 5: Risk of developing HOA owing to occupations in unskilled or basic labour
