# Supplementary material for: Physically demanding occupations among females and sex-related differences to develop osteoarthritis of the hip: a systematic review and meta-analysis
Source: J Occup Med Toxicol. 2024 May 6;19:14. doi: 10.1186/s12995-024-00415-8 (PMC11071200; doi:10.1186/s12995-024-00415-8)
Supplement: Supplementary file 2 — Supplementary Material 2. [file 12995_2024_415_MOESM2_ESM.docx]

## Female-dominated occupations

### Health Care

*Supplementary table S1: Association of the risk of HOA for women and occupations in health care*

| **Study** | **Occupational subgroups** | **Outcome** | **N**  (women/ men) | **n (%), IR (95%CI) per 100.000 PY** (women/ men) | **Effect (95 %-CI)** |
| --- | --- | --- | --- | --- | --- |
| **Akesson 1999** (CS) | | | | | |
| Exposure | Dental personal (dentists, dental assistants and dental hygienists) | New musculoskeletal symptoms in the hip during the last 7 days | 74 | 2 (2.7 %) | Women: RR: 2.1 (0.3; 17)  Men: n.d. |
| Reference | Medical nurses with varied and physically light work load (e.g. blood donor services) |  | 27 | 1 (3.7 %) |  |
| Exposure | Dental personal (dentists, dental assistants and dental hygienists) | New musculoskeletal symptoms in the hip during the last 12 months | 74 | 7 (9.5 %) | Women: RR: 0.8 (0.2; 2.8)*  Men: n.d. |
| Reference | Medical nurses with varied and physically light work load (e.g. blood donor services) |  | 27 | 3 (11.1 %) |  |
| **Andersen** **2012** (CS) | | | | | |
| Exposure | Health-care assistance | HOA | 411 580^a^ /84 898^a^ | 2753 (0.67 %)^a^, IR: 93.0^b^ /299 (0.35 %)^a^, IR: 75,6^b^ | Women: HR: 1.12 (1.07; 1.18)*^b^  Men: HR: 1.11 (0.99; 1.25)^b^ |
| Reference | Office worker |  | 594 773^a^ /316 543^a^ | 4468 (0.75 %)^a^, IR: 89.6^b^ / 1473 (0,47 %)^a^, IR: 60,2^b^ |  |
| **Elsner 1995** (CCS) | | | | | |
| Exposure | Nurses and kindergarten teacher | HOA | 18 | 7 (38.9 %)^a^ | Women: OR: 1.0 (0.35; 3.25)*^b^  Men: n.d. |
| Reference | All other women |  | 171 | 79 (46.2 %)^a^ |  |
| Exposure | Health care | HOA | 10 | 4 (40.0 %)^a^ | Women: OR: 1.4 (0.22; 9.74)^b^  Men: n.d. |
| Reference | All other women |  | 179 | 82 (45.8 %)^a^ |  |
| **Franklin 2010** (CCS) | | | | | |
| Exposure | Technicians and associate professionals (most frequent in women: office clerks, nurse assistants)^d^ | THR | 196/111^a^ | 67 (34.2 %)[hip]  7 (3.6 %) [hip and knee] /n.d. | Women: OR: 0.74 (0.44; 1.3)*^b^  Men: n.d. |
| Reference | Manager and professionals (most common in women: teachers, nurses; in men: teachers, doctors) |  | 115/211^a^ | 46 (40 %) /6 (5.2 %) [hip]  27 (12.8 %)/ 3 (1.4 %) [hip and knee] |  |
| **Hubertsson 2017** (CS) | | | | | |
| Exposure | Health care (nursing and midwifery professionals, personal care and related workers) | Sick leave due to HOA | 45 211 / 5077 | 216 (0.48 %)^a^ /28 (0.55 %) ^a^ | Women: OR: 1.18 (0.93; 1.50)^c^  Men: n.d. |
| Reference | Business and administration |  | 23 732 / 22 556 | 97 (0.41 %)^a^ / 76 (0.34 %)^a^ |  |
| Exposure | Health care | Disability pension due to HOA | 45 211 / 5077 | 51 (0.11 %)^a^ / 1 (0.02 %) | Women: OR: 6.91 (2.49; 19.13)*^c^  Men: n.d. |
| Reference | Business and administration |  | 23 732 / 22 556 | 4 (0.02 %)^a^ / 7 (0.03 %)^a^ |  |
| **Solovieva 2018** (CS) | | | | | |
| Exposure | Environmental officers and nurses | Full disability retirement due to HOA | 42 639^a^/ 5746^a^ | 50 (0.117 %)^a^,  IR: 14 (8; 27)^b^ / 11 (0.19 %)^a^,  IR: 24 (8; 78) | Women: HR: 2.99 (1.68; 5.32)*^c^  Men: HR: 3.80 (1.78; 8.10)^c^ |
| Reference | Professionals |  | 57 226^a^/ 77 573^a^ | 17 (0.030 %)^a^,  IR: 3 (1; 9)^b^ / 26 (0.034 %)^a^,  IR: 4 (2; 8)^b^ |  |
| *best correspondence to describe the association between occupations in health care and HOA  a: calculated by the authors  b: adjusted for age (1 quality point)  c: adjusted or matched for age and other important confounder (2 quality points)  d: most prevalent in men: office clerks, ship’s engineers  CCS: case-control study; CI: confidence interval; CS: cohort study; HOA: Hip osteoarthritis; HR: Hazard ratio; IR: incidence rate; N: number of persons; n.d.: no data; n: number of cases; OR: odds ratio; PY: person years; QS: quality score; RR: relative risk; THR: hip replacement therapy | | | | | |

### Cleaner

*Supplementary table S2: Association between the risk of HOA for women and occupations in cleaning*

| **Study** | **Occupational subgroups** | **Outcome** | **N** (women/ men) | **n (%), IR (95%CI) per 100.000 PY** (women/ men) | **Effect (95 %-CI)** |
| --- | --- | --- | --- | --- | --- |
| **Elsner 1995** (CCS) | | | | | |
| Exposure | Cleaner | HOA | 8 / 5 | 5 (62.5 %)^a^ / 3 (60 %) | Women: OR: 0.9 (0.21; 4.25)*^b^  Men: : OR: 0.9 (0.15; 5.67)*^b^ |
| Reference | All other women /men |  | 181 / 224 | 81 (44.7 %)^a^ / 131 (58.5 %) ^a^ |  |
| **Hubertsson 2017** (CS) | | | | | |
| Exposure | Cleaning (cleaners and domestic helpers) | Sick leave due to HOA | 8043 / 1642 | 22 (0.27 %)^a^ / 4 (0.24 %) | Women: OR: 0.66 (0.41; 1.07)^c^  Men: n.d. |
| Reference | Business and administration |  | 23 732 / 22 556 | 97 (0.41 %)^a^ / 76 (0.34 %)^a^ |  |
| Exposure | Cleaning | Disability pension due to HOA | 8043 / 1643 | 6 (0.07 %)^a^ / 10 (0.12 %) | Women: OR: 5.44 (1.50; 19.72)*^c^  Men: n.d. |
| Reference | Business and administration |  | 23 732 / 22 556 | 4 (0.02 %)^a^ / 7 (0.03 %)^a^ |  |
| **Solovieva 2018** (CS) | | | | | |
| Exposure | Building caretakers, cleaners, assistant nurses, and kitchen workers | Full disability retirement due to HOA | 43 761^a^/ 16 664^a^ | 194 (0.44 %)^a^, IR: 58 (44; 79)^b^/ 56 (0.336 %)^a^, IR: 43 (26; 75)^b^ | Women: HR: 3.29 (1.84; 5.91)*^c^  Men: HR: 2.58 (1.39; 4.80)*^c^ |
| Reference | Professionals |  | 57 226^a^/ 77 573^a^ | 17 (0.030 %)^a^, IR: 3 (1; 9)^b^/ 26 (0.034 %)^a^,  IR: 4 (2; 8)^b^ |  |
| **Vingard 1991** (CS) | | | | | |
| Exposure | Cleaners | Hospitalization due to OA | 7625 | 43 (0.56 %)^a^ | Women: RR: 1.20 (0.72; 1.45)*^b^  Men: n.d. |
| Reference | Low exposure blue-collar workers |  | 24 145 | 112 (0.46 %)^a^ |  |
| *best correspondence to describe the association between occupations in cleaning and HOA  a: calculated by the authors  b: adjusted for age (1 quality point)  c: adjusted or matched for age and other important confounder (2 quality points)  CCS: case-control study; CI: confidence interval; CS: cohort study; HOA: Hip osteoarthritis; HR: Hazard ratio; IR: incidence rate; N: number of persons; n: number of cases; OR: odds ratio; PY: person years; QS: quality score; RR: relative risk | | | | | |

### Sales

*Supplementary table S3: Association between the risk of HOA for women and occupations in sales*

| **Study** | **Occupational subgroups** | **Outcome** | **N** (women/ men) | **n (%), IR (95%CI) per 100.000 PY** (women/ men) | **Effect (95 %-CI)** |
| --- | --- | --- | --- | --- | --- |
| **Elsner 1995** (CCS) | | | | | |
| Exposure | Retail workers | HOA | 27 / 11 | 19 (70.4 %)^a^ / 5 (45.4 %)^a^ | Women: OR: 2.6 (0.95; 7.10)^b^  Men: OR: 0.5 (0.17; 1.59)*^b^ |
| Reference | All other women |  | 162 / 218 | 67 (41.3 %)^a^ /129 (59.2 %)^a^ |  |
| **Franklin 2010** (CCS) | | | | | |
| Exposure | Service and shop worker | THR | 246/66^a^ | 89 (36.2 %)(only hip), 8 (3.2 %) [hip and knee] /  28 (42.4 %) [hip], 3 (45.5 %) [hip and knee] | Women: OR: 0.79 (0.48; 1.3)^b^  Men: OR: 2.1 (1.0; 4.2)*^b^ |
| Reference | Manager and professional |  | 115/211^a^ | 46 (40 %) /6 (5.2 %) [hip]  27 (12.8 %)/ 3 (1.4 %) [hip and knee] |  |
| **Solovieva 2018** (CS) | | | | | |
| Exposure | Shop worker | Full disability retirement due to HOA | 28 052^a^ /13 216^a^ | 61 (0.22 %)^a^, IR: 28 (17; 50)^b^/ 24 (0.18 %)^a^, IR: 22 (10; 53)^b^ | Women: HR: 2.93 (1.61; 5.31)^c^  Men: HR: 2.35 (1.19; 4.67)*^c^ |
| Exposure | Customer service clerks |  | 17 953^a^/ 1724^a^ | 12 (0.07 %)^a^,  IR: 12 (5; 30)^b^/0 | Women: HR: 1.95 (0.98; 3.88)^c^  Men: n.d. |
| Reference | Professionals |  | 57 226^a^/ 77 573^a^ | 17 (0.030 %)^a^,  IR: 3 (1; 9)^b^/ 26 (0.034 %)^a^,  IR: 4 (2; 8)^b^ |  |
| *best correspondence to describe the association between occupations in sales and HOA  a: calculated by the authors  b: adjusted for age (1 quality point)  c: adjusted or matched for age and other important confounder (2 quality points)  CCS: case-control study; CI: confidence interval; CS: cohort study; HOA: Hip osteoarthritis; HR: Hazard ratio; IR: incidence rate; N: number of persons; n.d.: no data; n: number of cases; OR: odds ratio; PY: person years; QS: quality score; RR: relative risk; THR: total hip replacement therapy | | | | | |

### Gastronomy

*Supplementary table S4: Association between the risk of HOA for women and occupations in gastronomy*

| **Study** | **Occupational subgroups** | **Outcome** | **N**  (women/ men) | **n (%), IR (95%CI) per 100.000 PY**  (women/ men) | **Effect (95 %-CI)** |
| --- | --- | --- | --- | --- | --- |
| **Elsner 1995** (CCS) | | | | | |
| Exposure | Workers in hotels, gastronomy and households | HOA | 22 /14 | 12 (54.5 %)^a^ / 7 (50.0 %)^a^ | Women: OR: 1.4 (0.56; 3.56)^b^  Men: OR: 0.6 (0.18; 2.03)^b^ |
| Reference | All other women |  | 167 /215 | 74 (44.3 %)^a^ / 127 (59.1 %)^a^ |  |
| **Solovieva 2018** (CS) | | | | | |
| Exposure | Building caretakers, cleaners, assistant nurses, and kitchen workers | Full disability retirement due to HOA | 43 761^a^/ 16 664^a^ | 194 (0.44 %)^a^, IR: 58 (44; 79)^b^/ 56 (0.336 %)^a^,  IR: 43 (26; 75)^b^ | Women: HR: 3.29 (1.84; 5.91)^c^  Men: HR: 2.58 (1.39; 4.80)^c^ |
| Reference | Professionals |  | 57 226^a^/ 77 573^a^ | 17 (0.030 %)^a^, IR: 3 (1; 9)^b^/ 26 (0.034 %)^a^,  IR: 4 (2; 8)^b^ |  |
| **Vingard 1991** (CS, QS=13) | | | | | |
| Exposure | Waiters and hairdressers | Hospitalization due to HOA | 7243 / 2542 | 20 (0.27 %)^a^ / 8 (0.31 %)^a^ | Women: RR: 1.15 (0.83; 1.71)*^b^  Men: RR: 0.89 (0.42; 1.69)^b^ |
| Reference | Low exposure blue-collar workers |  | 24 145 / 91 057 | 112 (0.46 %)^a^ / 320 (0.35 %)^a^ |  |
| a: calculated by the authors  b: adjusted for age (1 quality point)  c: adjusted or matched for age and other important confounder (2 quality points)  CCS: case-control study; CI: confidence interval; CS: cohort study; HOA: Hip osteoarthritis; HR: Hazard ratio; IR: incidence rate; N: number of persons; n: number of cases; OR: odds ratio; PY: person years; QS: quality score; RR: relative risk | | | | | |

### Child care

*Supplementary table S5: Association between the risk of HOA for women and occupations in child care*

| **Study** | **Occupational subgroups** | **Outcome** | **N** (women/ men) | **n (%), IR (95%CI) per 100.000 PY** (women/ men) | **Effect (95 %-CI)** |
| --- | --- | --- | --- | --- | --- |
| **Elsner 1995** (CCS) | | | | | |
| Exposure | Nurses and kindergarten teacher | HOA | 18 | 7 (38.9 %)^a^ | Women: OR: 1.0 (0.35; 3.25)^b^  Men: n.d. |
| Reference | All other women |  | 171 | 79 (46.2 %)^a^ |  |
| **Hubertsson 2017** (CS) | | | | | |
| Exposure | Child care | Sick leave | 16.308 / 1209 | 53 (0.32 %) / 4 (0.33 %) | Women: OR: 0.77 (0.54; 1.08)^c^  Men: n.d. |
| Reference | Business/administration |  | 23 732 / 22 556 | 97 (0.41 %)^a^ / 76 (0.34 %)^a^ |  |
| Exposure | Child care | Disability pension | 16.308 / 1209 | 14 (0.09 %) / 0 | Women: 5.89 (1.92; 18.07)*^c^  Men: n.d. |
| Reference | Business/administration |  | 23 732 / 22 556 | 4 (0.02 %)^a^ / 7 (0.03 %)^a^ |  |
| **Solovieva 2018** (CS) | | | | | |
| Exposure | Teaching professionals | Full disability retirement due to HOA | 46 566^a^ /22 985 | 25 (0.050 %)^a^, IR: 6 (3; 14)^b^ / 6 (0.013 %)^a^, IR:3 (1;12)^b^ | Women: HR: 1.65 (0.87; 3.14)^c^  Men: HR: 0.64 (0.26; 1.58) |
| Reference | Professionals |  | 57 226^a^/ 77 573^a^ | 17 (0.030 %)^a^, IR: 3 (1; 9)^b^/ 26 (0.034 %)^a^,  IR: 4 (2; 8)^b^ |  |
| *best correspondence to describe the association between occupations in child care and HOA  a: calculated by the authors  b: adjusted for age (1 quality point)  c: adjusted or matched for age and other important confounder (2 quality points)  CCS: case-control study; CI: confidence interval; CS: cohort study; HOA: Hip osteoarthritis; HR: Hazard ratio; IR: incidence rate; N: number of persons; n.d.: no data; n: number of cases; OR: odds ratio; PY: person years; QS: quality score; RR: relative risk; THR: hip replacement therapy | | | | | |

### Hairdresser

*Supplementary table S6: Association between the risk of HOA for women and occupations in hairdressing*

| **Study** | **Occupational subgroups** | **Outcome** | **N** (women/ men) | **n (%), IR (95%CI) per 100.000 PY** (women/ men) | **Effect (95 %-CI)** |
| --- | --- | --- | --- | --- | --- |
| **Elsner 1995** (CCS) | | | | | |
| Exposure | Hairdresser | HOA | 6 | 4 (66.6 %)^a^ | Women: OR: 1.3 (0.25; 7.5)^b^  Men: n.d. |
| Reference | All other women |  | 183 | 82 (44.8 %)^a^ |  |
| **Vingard 1991** (CS, QS=13) | | | | | |
| Exposure | Waitresses or hairdresser | Hospitalization due to HOA | 7243 / 2542 | 39 (0.54 %)^a^ / 8 (0.32 %)^a^ | Women: RR: 1.15 (0.83; 1.71)*^b^  Men: RR 0.89 (0.42; 1.69) |
| Reference | Low exposure blue-collar workers |  | 24 145 / 91 057 | 112 (0.46 %)^a^ / 320 (0.35 %)^a^ |  |
| *best correspondence to describe the association between occupations in storage and transportation and HOA  a: calculated by the authors  b: adjusted for age (1 quality point)  c: adjusted or matched for age and other important confounder (2 quality points)  CCS: case-control study; CI: confidence interval; CS: cohort study; HOA: Hip osteoarthritis; HR: Hazard ratio; IR: incidence rate; N: number of persons; n.d.: no data; n: number of cases; OR: odds ratio; PY: person years; QS: quality score; RR: relative risk; THR: hip replacement therapy | | | | | |

## Male-dominated occupations

### Agriculture, fishery and forestry

*Supplementary table S7: Association of the risk of HOA for woman and men and occupations in agriculture, fishery or forestry*

| **Study** | **Occupational subgroups** |  | **Outcome** | **N**  (women/ men) | **n (%), IR (95%CI) per 100.000 PY**  (women/ men) | **Effect (95 %-CI95 %-CI)** |
| --- | --- | --- | --- | --- | --- | --- |
| **Andersen** **2012** (CS) | | | | | | |
| Exposure | Farmer |  | HOA | 55 132/ 161 923^a^ | 696 (1.26 %)^a^, IR: 103.7^b^/  3361 (2,1%)^a^, IR: 157,7^b^ | Women: HR: 1.22 (1.12; 1.33)^b^  Men: HR: 1.96 (1.84; 2.08)^b^ |
| Reference | Office workers |  |  | 595 685^/^ 316 543^a^ | 4468 (0.75 %)^a^, IR: 89.6^b^/  2475 (0,8%)^a^, IR: 60,2^b^ |  |
| **Franklin 2010 (CCS)** | | | | | | |
| Exposure | Farmers |  | THR | 242/273^a^ | 77 (31.8 %)/ 140 (51.3 %) [hip]/  18 (7.4 %)/ 10 (3,7 %)[hip and knee] | Women: OR: 0.62 (0.36; 1.0)^b^  Men: OR: 3.6 (2.1; 6.2)^b^ |
| Reference | Manager and professionals |  |  | 115/211^a^ | 46 (40 %) /6 (5.2 %) [hip]  27 (12.8 %)/ 3 (1.4 %) [hip and knee] |  |
| **Johansson 2018 (CS)** | | | | | | |
| Exposure | Farmer |  | Hip arthroplasty due to osteoarthritis | 24.284/72.852^a^ | 1061 (4.4 %)^a^/ 4298 (5.9 %) | Women: 1.40 (1.31; 1.49)^c^  Men: OR: 2.05 (1.98; 2.13)^c^ |
| Reference | All other occupations |  |  | 1.627.779^a^  / 1.782.581 ^a^ | 32.938 (2.0 %)^a^/30.535 (1.7 %)^a^ |  |
| **Solovieva 2018 (CS)** | | | | | | |
| Exposure | Agricultural and fishery workers |  | Full disability retirement due to HOA | 17 953^a^/ 34 477^a^ | 80 (0.45 %)^a^, IR: 57 (37; 93)^b^/ 135 (0.39 %)^a^, IR: 49 (35; 70)^b^ | Women: HR: 3.89 (2.04; 7.42)^c^  Men: HR: 1.92 (0.95; 3.89)^c^ |
| Reference | Professionals |  |  | 57 226^a^/ 77 573^a^ | 17 (0.023 %)a,  IR: 3 (1; 9)^b^/ 26 (0.034 %)^a^, IR: 4 (2; 8)^b^ |  |
| **Vingard 1991** (CS) | | | | | | |
| Exposure | Farmer |  | Hospitalization due to HOA | 1739 /35 981 | 12 (0.69 %) / 479 (1.3 %) | Women: RR: 1.47 (0.86; 2.86)^b^  Men: RR: 3.78 (2.91; 3.88)^b^ |
| Reference | Low exposure blue-collar workers |  |  | 24 145/ 91 057 | 112 (0.46 %)^a^/320 (0.35 %)^a^ |  |
| a: calculated by the authors  b: adjusted for age (1 quality point)  c: adjusted or matched for age and other important confounder (2 quality points)  CCS: case-control study; CI: confidence interval; CS: cohort study; HOA: Hip osteoarthritis; HR: Hazard ratio; IR: incidence rate; N: number of persons; n.d.: no data; n: number of cases; OR: odds ratio; PY: person years; QS: quality score; RR: relative risk; THR: hip replacement therapy | | | | | | |

### Craft work

*Supplementary table S8: Association between the risk of HOA for women and occupations in craft work*

| **Study** | **Occupational subgroups** | **Outcome** | **N**  (women/ men) | **n (%)**  (women/ men) | **Effect (95 %-CI)** |  |
| --- | --- | --- | --- | --- | --- | --- |
| **Elsner 1995** (CCS) | | | | | |  |
| Exposure | Occupations in textile industry | HOA | 13 / n.d. | 8 (61.5 %) | Women: OR: 1.2 (0.42; 3.90)  Men: n.d. | |
| Reference | All other women |  | 176 / n.d. | 78 (44.3 %) |  |  |
| **Franklin 2010** (CCS) | | | | | |  |
| Exposure | Craft worker and related trade workers  (most common for women: fish processing, sewers/for men: carpenters, construction workers) | THR | 196/236^a^ | 69 (35.2 %)[hip], 5 (2.5 %) [hip and knee] / 78 (33.1 %)[hip], 6 (2.5 %) [hip and knee] | Women: HR: 0.66 (0.39; 1.1)^b^  Men: HR: 1.5 (0.87; 2.7)^b^ |  |
| Reference | Manager and professional |  | 115/211^a^ | 46 (40 %) /6 (5.2 %) [hip]  27 (12.8 %)/ 3 (1.4 %) [hip and knee] |  |  |
| **Solovieva 2018** (CS) | | | | | |  |
| Exposure | Craft worker | Full disability retirement due to HOA | 4488^a^/ 11 492 | 16 (0.030 %)^a^, IR: 30 (12-79)^b^ / 33 (0.29 %), IR: 35 (18-75)^b^ | Women: HR: 2.99 ( 1.49; 5.99)^c^  Men: HR: 2.85 (1.58; 5.16)^c^ |  |
| Reference | Professionals |  | 57 226^a^ /77 573^a^ | 17 (0.030 %)^a^, IR: 3 (1; 9)^b^ / 26 (0.034 %)^a^, IR: 4 (2; 8)^b^ |  |  |
| a: calculated by the authors  b: adjusted for age (1 quality point)  c: adjusted or matched for age and other important confounder (2 quality points)  CCS: case-control study; CI: confidence interval; CS: cohort study; HOA: Hip osteoarthritis; N: number of persons; n.d.: no data; n.e. not estimated; n: number of cases; OR: odds ratio; QS: quality score; RR: relative risk; SHR: standardized hospitalization ratio (observed / expected number); THR: hip replacement therapy | | | | | |  |

### Construction worker

Supplementary table S9: Association of the risk of HOA for woman and occupations in construction workers

| **Study** | **Occupational subgroups** | **Outcome** | **N** (women/ men) | **n (%), IR (95%CI) per 100.000 PY**  (women/ men) | **Effect (95 %-CI95 %-CI)** |
| --- | --- | --- | --- | --- | --- |
| **Andersen 2012** (CS) | | | | | |
| Exposure | Construction worker | HOA | 38 485^a^/ 448.671^a^ | 139 (0.36 %)^a^,  IR: 93.8^b^ | Women: HR: 1.21 (1.03; 1.43)*^b^  Men: HR: 1.23 (1.15; 1.31)*^b^ |
| Exposure | Floor- and bricklayer |  | 96/4286^a^ | 0 | n.e. |
| Reference | Office worker |  | 595 685^a^ /316 543^a^ | 4468 (0.75 %)^a^, IR: 89.6^b^/  2475 (0,8%)^a^, IR: 60,2^b^ |  |
| **Solovieva 2018** (CS) | | | | | |
| Exposure | Construction worker, electricians and plumbers | Full disability retirement due to HOA | 2244^a^ /47 693^a^ | 3 (0.13 %)^a^  IR: 11 (2-81)^b^ | Women: HR 0.68 (0.14; 3.29)^c^  Men: HR: 1.70 (0.75; 3.84)*^c^ |
| Reference | Professionals |  | 57 226^a^/ 77 573^a^ | 17 (0.030 %)^a^,  IR: 3 (1; 9)^b^ |  |
| *best correspondence to describe the association between occupations in construction work and HOA  a: calculated by the authors  b: adjusted for age (1 quality point)  c: adjusted or matched for age and other important confounder (2 quality points)  CCS: case-control study; CI: confidence interval; CS: cohort study; HOA: Hip osteoarthritis; HR: Hazard ratio; IR: incidence rate; N: number of persons; n.e.: not estimated; n: number of cases; OR: odds ratio; PY: person years; QS: quality score; RR: relative risk; THR: hip replacement therapy | | | | | |

### Unskilled and basic level labour

*Supplementary table 11: Association between the risk of HOA for women and unskilled or basic level labour*

| **Study** | **Occupational subgroups** | **Outcome** | **N**  (women/ men) | **n (%), IR (95%CI) per 100.000 PY**  (women/ men) | **Effect (95 %-CI)** |
| --- | --- | --- | --- | --- | --- |
| **Franklin 2010** (CCS) | | | | | |
| Exposure | Operator and unskilled labour (building construction labourer, heavy truck and lorry driver, cleaner, factory work) | THR | 162/144^a^ | 46 (28.4 %) (only hip), 11 (6.8 %) [hip and knee] /  51 (35.4 %) (only hip), 6 (4.2 %) [hip and knee] | Women: OR: 0.60 (0.34; 1.1)^b^  Men: OR: 1.4 (0.78; 2.6)^b^ |
| Reference | Manager and professionals |  | 115/211^a^ | 46 (40 %) /6 (5.2 %) [hip]  27 (12.8 %)/ 3 (1.4 %) [hip and knee] |  |
| **Solovieva 2018** (CS) | | | | | |
| Exposure | Unskilled transport, construction, and manufacturing workers | Full disability retirement due to HOA | 6732^a^ / 18 388^a^ | 18 (0.27 %)^a^, IR: 39 (19; 84)^b^ / 86 (0.47 %)^a^, IR: 45 (29; 75)^b^ | Women: HR: 2.78 (1.33; 5.78)^c^  Men: HR: 2.94 (1.58; 5.47)^c^ |
| Reference | Professionals |  | 57 226^a^ /77 573^a^ | 17 (0.030 %)^a^, IR: 3 (1; 9)^b^ / 26 (0.034 %)^a^,  IR: 4 (2; 8)^b^ |  |
| a: calculated by the authors  b: adjusted for age (1 quality point)  c: adjusted or matched for age and other important confounder (2 quality points)  CCS: case-control study; CI: confidence interval; CS: cohort study; HOA: Hip osteoarthritis; HR: Hazard ratio; IR: incidence rate; N: number of persons; n.d.: no data; n: number of cases; OR: odds ratio; PY: person years; QS: quality score; RR: relative risk; THR: hip replacement therapy | | | | | |
